# Endoparasites of European Wildcats (*Felis silvestris*) in Greece

**DOI:** 10.3390/pathogens10050594

**Published:** 2021-05-13

**Authors:** Anastasia Diakou, Despina Migli, Dimitris Dimzas, Simone Morelli, Angela Di Cesare, Dionisios Youlatos, Petros Lymberakis, Donato Traversa

**Affiliations:** 1 School of Veterinary Medicine, Aristotle University of Thessaloniki, 54124 Thessaloniki, Greece; dimzas@vet.auth.gr; 2 School of Biology, Aristotle University of Thessaloniki, 54124 Thessaloniki, Greece; despmigk@bio.auth.gr (D.M.); dyoul@bio.auth.gr (D.Y.); 3 Faculty of Veterinary Medicine, University Teaching Veterinary Hospital, University of Teramo, 64100 Teramo, Italy; smorelli@unite.it (S.M.); adicesare@unite.it (A.D.C.); dtraversa@unite.it (D.T.); 4 Natural History Museum of Crete, School of Sciences and Engineering, University of Crete, Knossou Avenue, 71409 Irakleio, Greece; lyberis@nhmc.uoc.gr

**Keywords:** European wildcat, *Felis silvestris*, Greece, parasites

## Abstract

The European wildcat (*Felis silvestris*) is the only wild felid living in Greece. Wildcat populations are declining due to anthropogenic and phenological unfavourable conditions, and parasites may have an additional negative impact. In the present study, the occurrence of endoparasites in wildcats in Greece and the potential threats posed to wildcats, domestic animals, and humans in the study areas has been investigated. In a six-year period, 23 road-killed wildcats and 62 wildcat faecal samples were collected from different areas of the country. Necropsy for the detection of endoparasites and standard parasitological examinations of faecal samples were performed. Parasites were morphologically identified and, in selected cases, molecularly analysed. All necropsied wildcats (100%) were infected by three to 10 different parasite taxa, with the most prevalent being *Taenia taeniaeformis* (73.9%), *Toxocara cati* (60.9%), *Angiostrongylus chabaudi* (56.5%), *Ancylostoma tubaeforme* (39.1%), *Cylicospirura* spp. (34.8%), *Troglostrongylus brevior* (34.8%), and *Capillaria aerophila* (33.8%). Of the 62 faecal samples examined, 53 (85.5%) were positive for one or more parasite elements (larvae, eggs, or oocysts). The most frequent were *T. cati* (45.2%), *A. chabaudi* (29%), *C. aerophila* (24.2%), and Ancylostomatidae (17.7%). This is the first survey on endoparasites affecting wildcats in Greece. Some of the parasites here found are frequent in domestic and wild felids, while others, i.e., *Oslerus rostratus* and *Cylicospirura petrowi*, were described for the first time in the European wildcat. Most of them have a significant pathogenic potential, causing severe to hazardous diseases to infected felids and some, under specific circumstances, can also threaten human health.

## 1. Introduction

**Figure 1:**
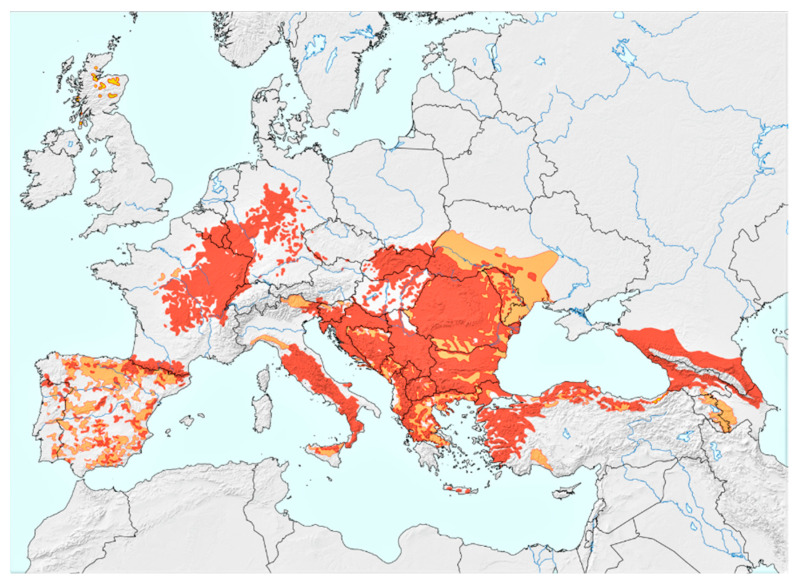
European wildcat (*Felis silvestris*) distribution. Coloured areas: red = present, orange = possibly present, yellow = possibly extinct. The map was kindly provided by Peter Gerngross, MSc [2]. (Reprinted with permission from ref. [2]. Copyright 2021 Gerngross, P. et al.).

The European wildcat, *Felis silvestris* (Carnivora, Felidae) is a small felid living in areas of Europe and Asia (Figure 1) [1,2]. Although it is listed as “Least Concern” by the International Union for Conservation of Nature and Natural Resources (IUCN), habitat loss and fragmentation as well as extensive hybridisation with domestic cats (*Felis catus*) have placed the species in an alerting conservation status [3]. These are important threats causing a decreasing population trend, which has already resulted in veiled extinction in some areas. In fact, an extended recent survey in different areas of Europe has revealed varying hybridisation levels in the examined animals and has shown possible extinction of the Scottish population [2,4]. The European wildcat is the only wild felid with confirmed reproducing populations in Greece and low levels of hybridisation, and it lives in several habitats including agricultural areas and wetlands [4,5].

Other factors threatening this felid species are human-caused mortality (road kills and poisoning) and competition with feral domestic cats for prey and territory [2]. The interaction with domestic cats in sympatric habitats may also result in disease transmission and both-ways-spill-over of pathogens, such as parasites [6,7]. Parasitism in wild animals is common, and its impact is studied under three perspectives: (i) the effect of infections/infestations on the wild host, (ii) the possibility of transmission and the impact to domestic animals, and (iii) the likelihood of implications on human health [8]. Even though parasites do not often cause life-threatening infections/infestations [8,9], they have the potential to undermine the wellbeing, health status, and overall fitness of wild animals, including the ability to prey, mate, and escape predators. Thus, under certain conditions, parasites are a significant threat for vulnerable populations [10].

Knowledge on parasites affecting European wildcats is scant, due to the elusive nature of these animals and minimum interactions with humans, which render challenging any sample collections. However, in the recent years, the scientific interest on parasites affecting wild felids has increased significantly, as novel insights have shed light on possible bridging infections with cardiopulmonary nematodes between wildcats and domestic cats [7,11,12,13,14]. Recent data have suggested that domestic cats in Greece may be infected by parasites and vector-borne pathogens, which could be shared by wildcats and in some cases have the potential to also infect dogs and humans [15,16,17,18,19,20,21]. Additionally, single reports of multiparasitism in two wildcats in Greece and Bosnia and Herzegovina have clearly proved that heavy parasitic infections may represent a factual threat for these animals [22,23]. Therefore, the aim of the present survey was to investigate in a focused epizootiological, large-scale study, the occurrence of endoparasites in wildcats in Greece by faecal and post-mortem examinations, and to assess the potential threats posed to wildcats, domestic animals, and humans in the study areas.

## 2. Materials and Methods

### 2.1. Study Areas, Animals, and Samples

**Figure 2:**
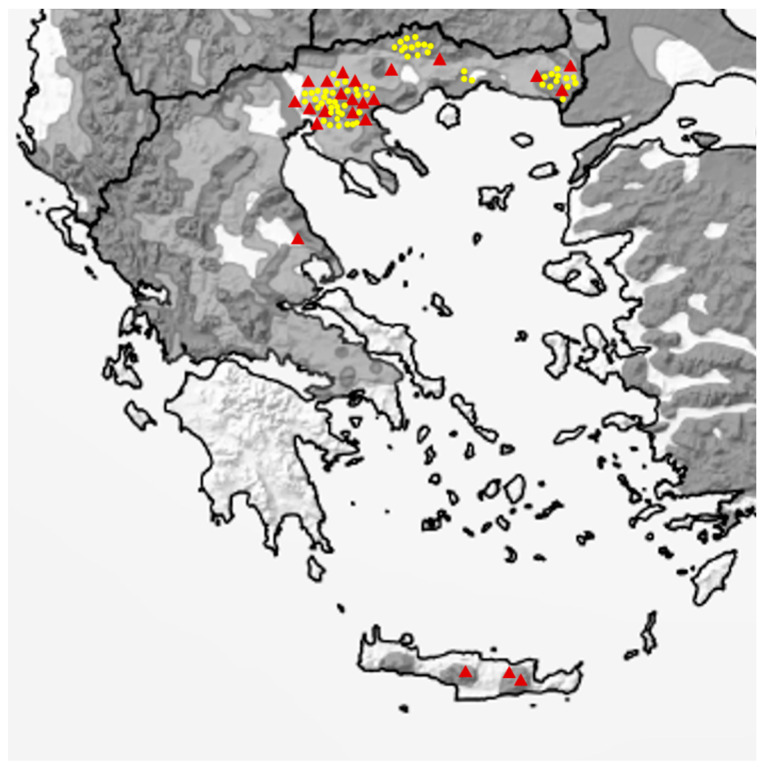
Map of Greece showing the European wildcat distribution areas (dark grey = present, light grey = possibly present, adapted from [2]) and the sampling spots (yellow dots: faecal samples, red triangles: road-killed animals). (Adapted with permission from ref. [2]. Copyright 2021 Gerngross, P. et al.).

From 2015 to 2021, road-killed wildcats and faecal samples were collected from different areas of Greece within the distribution range of *F. silvestris* (Figure 2). Wildcat presence was confirmed by sightings of animals and via camera traps, placed along trails, >1 km away from human settlements. Faeces were collected from wetlands, shrublands, and forests. Road-killed animals were found in different parts of the Greek mainland, while three specimens came from the Island of Crete.

**Figure 3:**
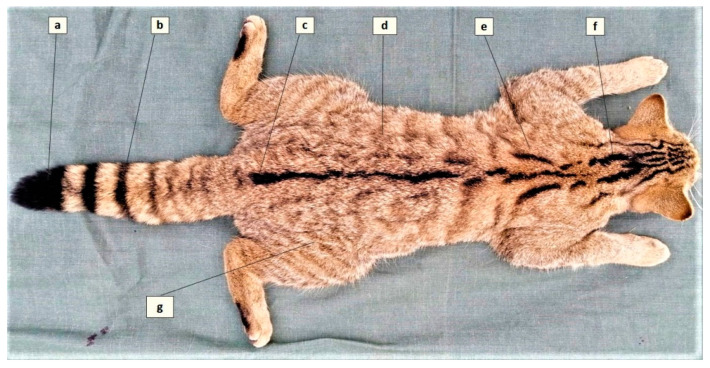
The seven pelage characteristics of the European wildcat (adapted from Kitchener et al., 2005). (**a**) Blunt tip of tail, (**b**) Distinct tail bands, (**c**) Dorsal line stopping at the base of tail, (**d**) Less than 25% broken stripes on flanks and hindquarters, (**e**) Two thick stripes on shoulder, (**f**) Four thick stripes on nape, (**g**) No spots on flanks and hindquarters.

Wildcats were identified according to the key proposed by Kitchener et al. (2005) [24]. Accordingly, seven pelage characters (Figure 3) were assessed, each contributing with a score (1 = domestic, 2 = hybrid, 3 = wildcat) that enables the differentiation of wildcats (score of >19, inclusion criterion in the present study) from domestic cats and hybrids.

**Figure 4:**
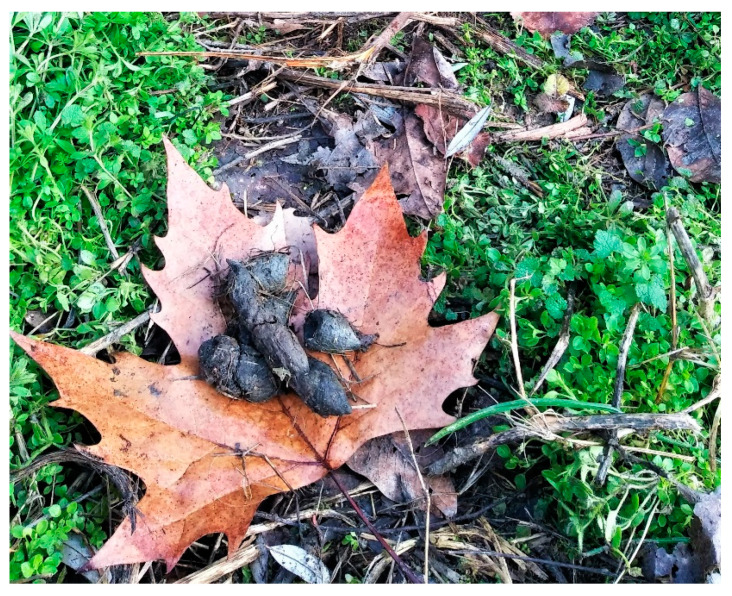
Faeces of wildcat. Note the concave and convex surfaces of the pieces.

**Figure 5:**
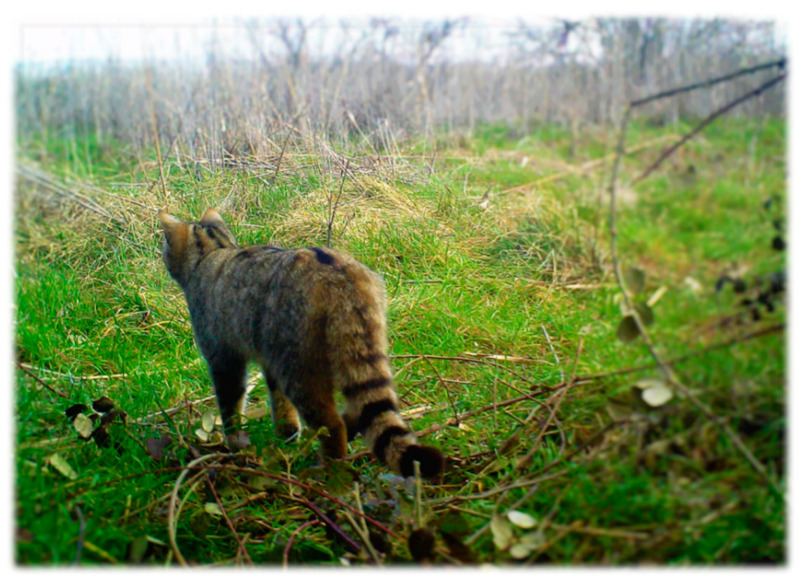
European wildcat trapped by the camera system set in the areas of the study.

Wildcat faeces were identified by the spot where they were found, i.e., on a path border, in thick grass or aside small bushes, and by their morphological characteristics [25], i.e., dark colour, the presence of several pieces with smooth and shiny surface, tips between the pieces of the same dropping concave on one side and convex on the other (Figure 4). In most cases, short dark hair, and small bones, most likely of rodent origin, were components of the faeces, along with plant material. Faecal samples were collected individually, tagged with date and coordinates, and kept frozen until examination. Moreover, the origin of faecal samples was confirmed by camera trapping, which was used to indicate the areas where only wildcats were captured (Figure 5).

The examined faecal samples and carcasses have been genetically analysed in a parallel, ongoing study on the genetic profile of wildcats in Greece, conducted by three of the authors (D.M., D.Y., P.L), and their wildcat origin was confirmed (unpublished data). The whole survey was carried out within the framework of a long-term project, focusing on the ecology, spatial movement, and genetic analysis of wildcat populations, which is a collaboration with the School of Biology Aristotle University of Thessaloniki, the Forest Research Institute of Thessaloniki, the Capel Manor College of London, and the Royal Zoological Society of Scotland [26].

### 2.2. Post-Mortem Examination

Only fresh carcasses, in good condition and with closed abdomen and thorax were collected and stored at −20 °C until they were transferred to the Laboratory of Parasitology and Parasitic Diseases, School of Veterinary Medicine, Aristotle University of Thessaloniki, Thessaloniki, Greece. In addition, frozen carcases of wildcats kept in the collection of the Natural History Museum of Crete were included in the study. Before examination, the carcases were left overnight to thaw in room temperature. The post-mortem parasitological examination included the inspection of the skin, eyes, mouth cavity, respiratory system, heart and pulmonary arteries, stomach, intestines, and kidneys. The respiratory tract (trachea, bronchi, and bronchioles), lung parenchyma lesions (nodules, emphysemas), heart and pulmonary arteries, intestines, gallbladder and bile ducts, and urinary bladder were opened and examined under a stereomicroscope. Furthermore, contents of the airways, lung vessels, and gastrointestinal tract were washed by tap water, and the sediment was examined under stereomicroscope and light microscope. The parasites found were washed in saline solution and, depending on their size, were examined under the stereomicroscope or temporarily mounted on slides and examined under an optical microscope. Finally, a faecal sample from the rectum was collected and examined as described below.

### 2.3. Faecal Examination

Faeces collected from the field and from the rectum of the dead animals were examined by two conventional concentration methods, i.e., ZnSO_4_ centrifugation flotation [27] and Telemann sedimentation [28].

### 2.4. Morphological Identification of Parasites

All parasites (adults, larvae, eggs, cysts, and oocysts) retrieved were identified based on the location of parasitism and their morphological and morphometric characteristics. Adult parasites were identified according to their general morphology at the Phylum level, and then according to (i) size, body shape, morphology, and arrangement of internal organs for trematodes, (ii) morphology of scolex, strobila, and mature proglottids for cestodes, (iii) size, cuticle structures (e.g., cervical alae, bursa copulatrix, papillae), anterior and posterior end, oesophagus size and morphology, reproductive organs of males, uterus, and vaginal opening for females for nematodes, and (iv) size, body shape, proboscis morphology, hooks number and arrangement, and internal organ morphology for acanthocephalans. Similarly, at faecal examinations, the identification was based on length, width, posterior and anterior end for larvae, size, shape, colour, shell thickness, surface morphology and content (zygote, blastomeres, larva) for eggs, and size, shape, and content for protozoa (cysts or oocysts). All morphological identifications were based on keys and features published in the international literature [29,30,31,32,33,34,35].

### 2.5. Molecular Identification of Parasites

Selected adult parasites were preserved in alcohol 70% and then further examined by molecular methods to confirm the morphological identification. This additional examination was based either on the recent evidence of taxonomical, biological, and epizotiological unresolved issues for some cardio-respiratory nematodes, as for *Angiostrongylus* spp., *Aelurostrongylus abstrusus*, *Troglostrongylus* spp. [7,13], or the necessity to corroborate the microscopic data for poorly known nematodes, such as *Cylicospirura* spp.

A representative number of adult nematodes morphologically identified as *A. chabaudi* and *T. brevior* (4 and 5 adult worms, respectively), and larvae of *A. chabaudi*, *T. brevior* and *A. abstrusus* were subjected to a diagnostic triplex PCR specific for diagnostic markers within the rDNA ITS2 of these nematodes [36]. A nematode morphologically identified as *Cylicospirura* spp. was subjected to a PCR-based assay specific for the mitochondrial gene encoding for the *cox*1 subunit b using a Spirurida-universal primer set [37].

The amplicons were purified and sequenced, and sequences were determined in both directions and the electropherograms verified by eye with the software Chromas Lite. The sequences were aligned using the software program DAMBE and compared with those available in the GenBank™.

## 3. Results

Overall, 23 wildcat carcasses and 62 faecal samples of wildcats were examined. The results of all examinations are shown in Table 1. Parasite elements found as a clear (e.g., *Eimeria* oocysts) or probable (e.g., *Dicrocoelium dendriticum* eggs) result of pseudoparasitism have been excluded from the results.

### 3.1. Necropsy

Parasites were found in all 23 necropsied wildcats (100%). The findings of necropsy were supplemented with those of the faecal examinations, which in many cases showed additional infections with protozoan and metazoan parasites. The most prevalent parasite found was the cestode *Taenia taeniaeformis* (17/23, 73.9%), followed by the nematodes *Toxocara cati* (14/23, 60.9%), *Angiostrongylus chabaudi* (13/23, 56.5%), *Ancylostoma tubaeforme* (9/23, 39.1%), *Cylicospirura* spp. (8/23, 34.8%), *Troglostrongylus brevior* (8/23, 34.8%), and *Capillaria aerophila* (7/23, 33.8%), while 17 more parasite taxa were also found (Table 1). Mixed infections were documented in all necropsied animals with the highest parasite diversity recorded in one animal with 10 different parasites. Nematodes, represented by species that inhabit both the gastrointestinal tract and cardio-pulmonary system, were observed with the highest diversity of species and mixed infections in most animals. Lists of the cases of polyparasitism are presented in Table 2 and Table 3.

**Figure 6:**
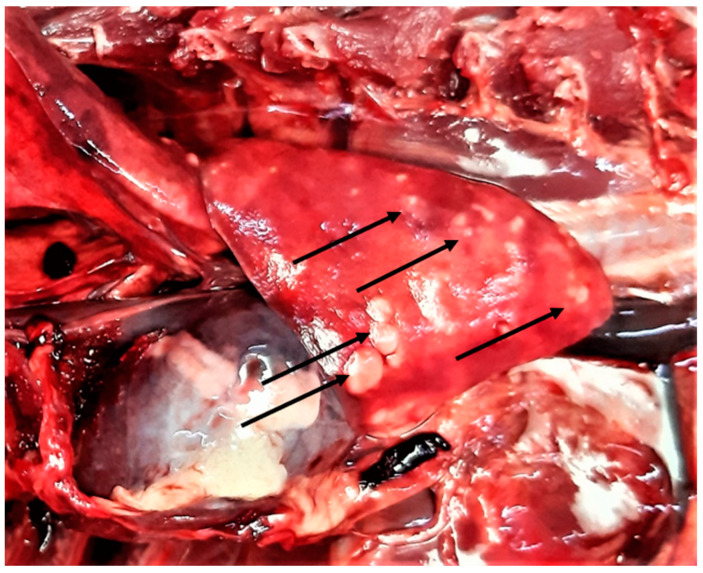
Lung gross lesions (arrows) in a wildcat with a mixed infection of five cardio-pulmonary parasites, i.e., *Aelurostrongylus abstrusus*, *Troglostrongylus brevior*, *Capillaria aerophila*, *Angiostrongylus chabaudi*, and *Dirofilaria immitis*.

In general, no lesions compatible with the parasitic infections were found on gross examination of the organs except for the lungs, whereby all cases of lungworms resulted in verminous bronchopneumonia (Figure 6).

### 3.2. Faecal Examinations

Of the 62 faecal samples examined, 53 (85.5%) were positive for one or more parasite elements (larvae, eggs, or oocysts). The most prevalent finding was *T. cati* in 28 (45.2%) samples, followed by *A. chabaudi* in 18 (29%), *C. aerophila* in 15 (24.2%), and Ancylostomatidae in 11 (17.7%) samples. The protozoa found were Coccidia, with the most prevalent being *Sarcocystis* spp. in 10 (16.1%) and *Cystoisospora felis* in 8 (12.9%) samples. Other findings included trematodes, i.e., *Alaria alata* and *Opisthorchis felineus*, cestodes i.e., *Mesocestoides* spp., and Diphyllobothriidae and Taeniidae eggs (Table 1). Some Capillariidae and trematode eggs found in faecal examinations could not be identified to a lower taxon and were equally considered true infections or pseudoparasitism.

Representative gross post-mortem findings, adult parasites, and microscopic findings (eggs, oocysts, sporocysts) are shown in Figure 6, Figure 7, Figure 8, Figure 9, Figure 10, Figure 11, Figure 12, Figure 13 and Figure 14.

### 3.3. Molecular Identification of Selected Parasites

The diagnostic triplex PCR confirmed the morphological identification of adult *A. chabaudi* and *T. brevior* and of *A. chabaudi*, *T. brevior*, and *A. abstrusus* larvae. The sequence generated by the nematode morphologically identified as *Cylicospirura* spp. displayed 100% homology with the *cox*1 sequence Accession Number KF719952.1 of *Cylicospirura petrowi*.

## 4. Discussion

This is the first epizootiological survey on endoparasites affecting wildcats in Greece and one of the most complete studies on the parasitic fauna of a large number of these elusive animals. In fact, the inherent difficulties in these surveys are reflected on the small number of parasitological data on wildcats globally. Indeed, current knowledge comes from restricted geographic areas, a few countries, and it derives from a small sample size [38,39,40,41], specific groups of parasites [11,42], faecal examinations only [43], combination of faecal examination and examination of stomach and intestine contents [44,45], or finally, single case reports [22,23,33,46,47]. In the present study, a combination of faecal examination of field-collected samples, with necropsy and copromicroscopic examination of road-killed animals found in the same areas, provided a comprehensive picture of parasitic fauna and infection rates in wildcats in Greece.

The fact that all necropsied animals were infected and that polyparasitism was universal is not surprising and in accordance with previous surveys [45]. Negative results for a few faecal samples could be attributed to the intermittent shedding of eggs, larvae, cysts, and oocysts in the faeces, and/or to environmental conditions that may have affected the conservation of the parasite developmental stages. Furthermore, the occurrence of metastrongyloid larvae in the faeces collected from the field may be underestimated, as the Baermann method was not applicable in frozen samples. Finally, in some cases where necroscopic findings were not in accordance with faecal examination in the same animal, immature parasites (prepatency), single sex parasitism, or intermittent shedding of eggs or larvae could be the reason. On the other way around, when only eggs or larvae were found, the failure to detect the corresponding adult parasites is attributed to their location (parenchyma) or strong adhesion in a location hard to reach.

### 4.1. Protozoa

The intestinal protozoa found in the faecal examinations have been reported previously in wildcats [45] and are common in domestic cats. *Cystoisospora* spp. is reported in wildcats with 4.1–29.4% positivity rates [43,44,45] and have a direct life cycle. The animals get infected by ingesting the sporulated oocysts from the environment. However, rodents and other small vertebrates can act as paratenic hosts [48]. In some cases, and especially in young animals and heavy infections, these parasites may cause severe enteritis with villi necrosis, diarrhoea, and even death [48]. Genera of the Toxoplasmatinae subfamily i.e., *Toxoplasma*, *Besnoitia*, and *Hammondia*, share oocyst morphology (<16 μm in diameter) and cannot be morphologically differentiated. These organisms as well as the coccidian *Sarcocystis*, have an indirect life cycle, and felids get infected by consuming intermediate hosts that carry the parasitic cysts in their tissues [49,50]. Various species of mammals are intermediate hosts of all four genera, including prey of wildcats and livestock. Felids are definitive hosts of *Toxoplasma gondii*, a parasite of great relevance in animal and human medicine, causing severe disease in immunocompromised individuals and abortions [50,51]. However, the whole range of species of *Besnoitia*, *Hammondia*, and *Sarcocystis* having felids as definitive host is yet to be determined [49,52,53].

### 4.2. Trematodes

This is the first record of *A. alata* in wildcats in Greece, and it confirms previous data indicating its occurrence in canids in the country [40,49,54,55]. The adults were well embedded in the mucosa of the intestine, and in all cases, they were located in the small intestine just before the caecum. This trematode has a complex life cycle with two intermediate hosts. Carnivores get infected by ingesting the mesocercariae, which is an interjectional stage between the cercariae and the metacercariae, found in the second intermediate host (amphibians) or in paratenic hosts [56]. The clinical impact of alariosis in definitive hosts is unclear due to the rarity of this parasitosis in domestic carnivores. However, respiratory and/or systemic signs during the mesocercariae migration and enteritis in case of heavy intestinal infection have been reported [57]. The second trematode found, *O. felineus*, infects wildcats when they consume the second intermediate host, i.e., freshwater fishes, mainly cyprinids. For wildcats living in wetlands (i.e., most animals here examined), fish represents a part of their diet. In the case of heavy parasitic load, the adult stages living in the bile ducts, gallbladder, and small intestine of the felid host cause chronic inflammation, leading to liver failure [58]. Regarding the impact of these trematodes in human health, alariosis is an emerging food-borne parasitosis that has been proven fatal. Humans get infected by consuming uncooked meat of a second intermediate (e.g., frogs) or a paratenic host (e.g., wild boar) [56]. On the other hand, *Opisthorchis* spp. is a well-known agent of human foodborne hepatic trematodosis with most cases reported form areas of the eastern European and Baltic countries [59], but now increasingly described in other European countries, including Italy [60]. Human opisthorchiosis ranges from a subclinical infection to possible hepatic neoplastic transformation [61].

### 4.3. Cestodes

The cestode *T. taeniaeformis*, a cosmopolitan taeniid of felids with rodents as the intermediate host, was the most frequent parasite found in the present survey. This corroborates data from previous studies indicating the frequent occurrence of this endoparasite in wildcats [38,45,62]. Accordingly, the stomach content (when present) of the wildcats here necropsied was constituted mostly of rodents, thus confirming a well-established transmission route for this tapeworm. Only four of the 17 animals positive for *T. taeniaeformis* were copromicroscopically positive for taeniid eggs, and the positivity percentage in faecal samples collected from the environment (1.6%) was very low compared to the infection rate at necropsy (73.9%). This confirms that in most cases, infected animals shed only a few eggs and rather release mature proglottids. Thus, copromicroscopic analysis is considered unreliable to diagnose cestodes in felines. While this is true for Cyclophyllidea cestodes [63,64], it does not occur in Diphyllobothriidea because eggs of the latter parasites are shed while proglottids are still attached to the strobila within the host [64]. Accordingly, all animals (17.4%) found infected by adult *Spirometra* spp., scored positive for diphyllobothrid eggs in the faeces, and the percentage of these eggs in the faecal samples collected from the field was at a similar prevalence level (12.9%). The source of infection of wildcats with *Spirometra* spp. and other tapeworms (e.g., *Mesocestoides* spp.) here found is again rodent prey, i.e., the main second intermediate host [64]. Even though the clinical impact of adult cestodes is considered minor in felines [65], sometimes, they can have an important mechanical and traumatic impact to the intestine [66]. This is especially true for *T. taeniaeformis*, as it is the most robust felid cestode, provided with a double row of hooks [67]. The cestodes found here may also infect domestic cats [68]; thus, free roaming and hunting domestic cats living in areas of wildcat distribution are at higher risk of such infections. Furthermore, the larval stage (plerocercoid) of *Spirometra* spp. may cause a severe parasitic disease (i.e., sparganosis) in humans, which is characterised by various symptoms, depending on the final location of the larva, that can migrate systemically, with the most dramatic impact in the central nervous system [69].

### 4.4. Gastrointestinal Nematodes

The felid ascarid *T. cati* was the most prevalent intestinal nematode found in the examined wildcats, followed by hookworms, which were all identified as *A. tubaeforme* based on adult stage morphology, and the stomach worms *Cylicospirura* spp. and *Physaloptera* spp. Indeed, *T. cati* is the most common intestinal nematode also in domestic cats due to its highly efficient life cycle and multiple modes of infection (e.g., ingestion of environmental infective eggs, preying of paratenic hosts harbouring larvae, or lactogenic route) [70]. Many felines are subclinically infected, although clinical ascaridosis occurs in the case of high parasitic load, causing pneumonia during larval migration and enteritis by adult worms. Occasionally, fatal outcome may occur due to intestinal obstruction, rupture, and subsequent peritonitis [70,71]. The hookworm *A. tubaeforme* infects felids mainly via the ingestion of L3 from the environment or in paratenic hosts (prey) and to a lesser extent by their percutaneous penetration. This is a common and cosmopolitan parasite of domestic cats although not so prevalent as *T. cati* in Greece, as shown in previous studies [15,19,20]. Adult parasites play a major pathogenic role, as they injure the mucosa of the small intestine, ingest blood, and cause lesions of the intestinal epithelium. Enteritis, dehydration, weight and blood loss, anaemia and even death may occur [70,71]. Nematodes of the genera *Toxocara* and *Ancylostoma* are associated with human infections. The canid species *Toxocara canis* and *Ancylostoma canimum* are confirmed zoonotic parasites, causing visceral and ocular *larva migrans* and cutaneous lesions, respectively. The role of the corresponding felid species, i.e., *T. cati* and *A. tubaeforme* is yet to be clarified, though it is likely underestimated, especially in the case of *T. cati* [71,72]. Infected canids and felids shed eggs in the environment, from where domestic animals may get infected and further spread these zoonotic agents in synanthropic settings [73,74]. The risk posed by parasites harboured by wildlife for pets and people is an emerging threat attributed to urbanisation and changes in land use. This can occur for wildcats and free ranging or feral cats, as in the case of other wild animals, e.g., raccoons, spreading the ascarid *Baylisascaris proyconis*, a parasite of major zoonotic importance [75].

**Figure 8:**
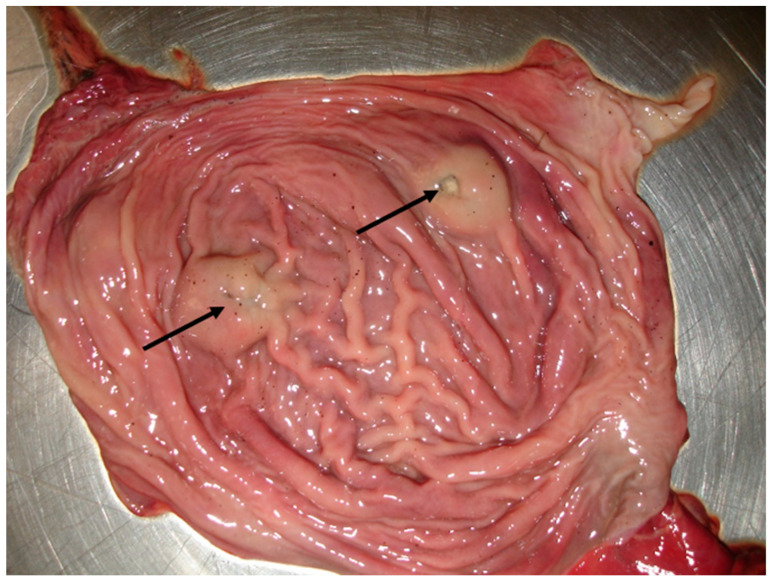
Gastric nodules caused by the stomach nematode *Cylicospirura* spp. in a wildcat. The arrows indicate the opening of the nodule from where the eggs of the parasite fall into the gastric lumen.

Both stomach worms found herein, i.e., *Cylicospirura* spp. and *Physaloptera* spp., have an indirect life cycle with various insects, e.g., beetles and crickets, as intermediate hosts and small vertebrates as paratenic hosts. *Cylicospirura* spp., a nematode frequently found in this study and in other wild felids [76], is only rarely described in domestic cats [77]. In the European wildcat, *Cylicospirura* spp. infection was previously detected in two faecal samples (eggs) in Italy [43] and in one necropsied animal in Germany [38]. The present data indicate the first molecularly confirmed report of *C. petrowi* (formerly known as *Petrowospirura petrowi*) [76] in Europe. The presence of these nematodes within the gastric wall causes the formation of nodules where the adults live in a ramified burrow. Accordingly, all carcasses positive for *Cylicospirura* spp. had one to three gastric nodules (Figure 8). Conversely, *Physaloptera* spp. live free in the gastric lumen, causing gastritis accompanied with vomiting, weight loss, and melena in both wild and domestic canids and felids [78]. It is important to note that the occurrence of *Cylicospirura*-like or *Physaloptera*-like larvated eggs in wild felid faeces should be evaluated conservatively as for their actual origin, as many spirurids, with often indistinguishable egg morphology, are harboured by rodents, birds, hedgehogs, and other felid prey, resulting to pseudoparasitism in examined predators [79].

### 4.5. Extra Intestinal Nematodes

Cardio-pulmonary nematodes were very common in the examined animals. The most prevalent parasite was *A. chabaudi* followed by *A. abstrusus*, *T. brevior*, and *C. aerophila* (syn. *Eucoleus aerophilus*). The respiratory nematode *Oslerus rostratus* and the heartworm *Dirofilaria immitis* were less prevalent, as they were found in one animal each. Interestingly, one wildcat had a mixed infection of five cardio-pulmonary parasites (all but *O. rostratus*), showing the most severe gross pulmonary lesions (Figure 6), and four animals had a mixed infection with four parasites (Table 2, animal n. 12).

*Angiostrongylus chabaudi* is a nematode of the pulmonary arteries and right chambers of the heart of wildcats, which is shown to be the natural definitive hosts after the first patent infection described in a wildcat in Greece [33]. Since then, the parasite was found in single case reports from Eastern Europe [23,46,47], in an epizootiological study in Italy [11], and in a live wildcat captured and hospitalised in Greece [12]. In the present study, *A. chabaudi* was the most prevalent extra-intestinal nematode, found in over half of the necropsied animals.

The cat lungworm *A. abstrusus* has been described in many wild felid species, though confirmed cases are few [7,80]. This parasite unequivocally infects European wildcats, as proven by epizootiological surveys and case reports [11,12,23]. In the present study, adult stages were not found in all necropsied animals shedding L1, which was probably due to the localisation of the parasites (i.e., pulmonary parenchyma). However, all infected wildcats showed pulmonary gross lesions compatible with aelurostrongylosis. Conversely, one wildcat was positive for *T. brevior* adults but negative for L1 at the faecal examination. This could be attributed to the use of flotation instead of the Baermann method or to the intermittent release of L1 by the adult parasites.

The capillarid *C. aerophila* infects mainly wild carnivores, including foxes and mustelids (considered the reservoir hosts) and wildcats with prevalence rates up to 33.3% [11,38,42,43,81], while it is less frequent in domestic cats [82]. The identification of capillarid eggs at faecal examination should be based on a careful appraisal of morphological and morphometric features [17]. Other than *C. aerophila*, three morphologically different capilariid eggs were found herein, which were compatible with species affecting carnivores, i.e., *Capillaria* (*Eucoleus*) *boehmi* (inhabiting the nose nasal cavity and paranasal sinuses of canids), *Capillaria* (*Aonchotheca*) *putorii* (a gastric parasite of felids), and *Capillaria hepatica* (syn. *Calodium hepaticum*), which is a rodent species that is occasionally recorded in carnivores. However, as these eggs were found in field-collected faeces, the origin of parasitism should remain suggestive due to the large number of Capillariidae species infecting wildlife (e.g., birds, mammals, and fish) which can be preyed by wildcats [83,84].

A single female *O. rostratus* was found in one carcass partially embedded in the bronchial epithelium, while no larvae were found in the faeces, which was likely due to the absence of male nematodes. The lack of *O. rostratus* L1 in some infected animals has also been attributed to the encapsulation of the parasite in a strong fibrous pseudo-cystic formation during a regressive phase of infection [85]. However, in the present case, the parasite was only half embedded in the bronchial tissue, with its posterior end, i.e., the part where the vulva is located, free in the bronchus lumen. To the best of the authors’ knowledge, this is the first report of this parasite in European wildcat. Τhe epizootiology of the parasite is obscure, although its life cycle is similar to those of other Metastrongyloidea, with gastropods as intermediate hosts. The parasite is rare and sporadic in domestic cats, even in areas where other metastrongyloids are enzootic [7,86]. However, *O. rostratus* has been found with high prevalence (up to 60%) in domestic cats from Sri Lanka and Majorca Islands [87,88]. The clinical impact of *O. rostratus* is also unknown, although inflammation with fibrosis, hyperplasia, and hypertrophy of the mucosa has been described at the point of encapsulation in the bronchial tissue [85].

The heartworm *D. immitis*, transmitted by mosquitoes, has domestic dogs as the main reservoir hosts, although felids can be infected, especially in hyperenzootic areas [22,89]. Accordingly, the infected wildcat was found in the area where the infection prevalence in domestic cats was recently found to be 9.4% [22]. As unsuitable hosts, felids are usually infected with a few or/and immature worms [90], as in the present case, where only one adult male was found. This is the third record of *D. immitis* in a wildcat, after the descriptions in Serbia and Romania [91,92].

All cardio-pulmonary nematodes may seriously impair the health of infected animals and, especially in cases of multiple parasitism, this may be life-threatening [12,23]. These infections may reduce animal fitness, rendering it vulnerable in various circumstances, e.g., vehicle collisions, territory fighting, and prey hunting. In enzootic areas where wildcats and domestic cats live in sympatry, bridging infections may occur, and similar cases of polyparasitism have been also reported in domestic cats [93,94]. The spill-over of these parasites between wildlife and domestic animals is an important factor shaping their epizootiology and determining their role in feline medicine. For example, *T. brevior*, a parasite of wildcats [11,42] (present results), is now recognised as an important agent of parasitic bronchopneumonia in domestic cats, with areas of occurrence overlapping those of wildcat distribution [7,95]. Specific biological traits of *T. brevior* may have contributed to its successful transmission between felid species. The ability of *T. brevior* to increase its developmental rate during hibernation of the intermediate gastropod host, and ensure abundance of infective larvae in springtime, in combination with its ability of vertical transmission, set the conditions for (a) the infection of the highest number of offspring possible, and (b) the survival and maintenance of the infection in domestic cat populations, even in the absence of the natural host [96].

Conversely, *A. chabaudi*, despite its high prevalence in wildcats, has been only sporadically found in domestic cats and never in patent infections [93,94]. To date, it is not considered an important pathogen in feline parasitology, most probably because of its better adaptation in wildcats [13]. Finally, *O. rostratus* according to all data so far is a rare parasite both in wildcats and domestic cats, and it is probably better adapted to specific felid species, e.g., lynxes [7,85], that are, at least currently, not resident in Greek territory. Nevertheless, it is important to remain vigilant in order to evaluate a potential future spreading of these parasites into other felid populations.

Of these extra-intestinal parasites, *C. aerophila* and *D. immitis* have a confirmed zoonotic importance. The former has been reported in humans only rarely [97], while *D. immitis* is increasingly documented in people presenting pulmonary granulomas and lesions in other tissues and organs [90,98]. According to the prevalence of infection in wildcats found here and in previous studies, these animals are major spreaders of *C. aerophila* in the environment. Conversely, their contribution to the maintenance and spreading of *D. immitis* via the infection of mosquitoes is very limited to nil, as most infected felines are not microfilariaemic [90].

The factual distribution of *T. callipaeda* in wildcats of Greece may be underestimated based on the present results because the eyes were poorly conserved in most carcasses. Animals get infected when the fruit fly, *Phortica variegata* carrying L3, feeds on lachrymal secretions [99]. The role of wildlife in the epizootiology of this parasite is important as wild animals represent reservoirs that facilitate the expanding distribution and prevalence of ocular thelaziosis [100]. The impact of this nematode may be significant both for wildlife and domestic animals, as it can cause severe eye damage including keratitis, corneal opacity, and/or ulceration, which combined with mechanical injury and secondary infections of the eye may result in sight impairment or sight loss [101]. Importantly, cases of human thelaziosis due to *T. callipaeda* are increasingly reported, especially in areas where the prevalence of animal infection is high [102].

### 4.6. Acanthocephalans

A specimen of the genus *Centrorynchus* was found in the intestine of one animal. Acanthocephala have been previously described in European wildcats, both in necropsy and in copromicroscopic examinations [39,43]. The eggs of different acanthocephalan species have similar morphology and their presence in wildcat faeces may be attributed to actual infection or to pseudoparasitism, being frequent parasites of insectivorous animals (e.g., birds, hedgehogs), which are common prey of wildcats [6]. Acanthocephala have an indirect life cycle with arthropod intermediate hosts, while felids typically get infected by consuming small vertebrates that serve as paratenic hosts [64]. Postcyclic transmission is an interesting phenomenon occurring in Acanthocephala, relevant to carnivore parasitism. In this case, when a carnivore preys on animals that carry adult Acanthocephala, the parasites may survive and establish in the intestine of the predator [103]. This predator–prey aspect of acantocephalan transmission, depending on both the role of paratenic hosts and the postcyclic transmission, may be the reason of the rare occurrence of these parasites in domestic cats compared to wildcats and other carnivores [6]. In heavy parasitism, the injury that the acantocephalans cause on the intestinal wall with their thorny proboscis may result in severe enteritis and even penetration of the intestinal wall, which is accompanied by peritonitis [104].

### 4.7. Ticks

The presence of Ixodidae ticks in three wildcats shows the potential of pathogens transmission of veterinary and human medicine importance. These pathogens include the bacteria, *Ehrlichia* spp., *Borrelia* spp., *Anaplasma* spp., and *Rickettsia* spp., and the haemoprotozoa *Hepatozoon* spp. and *Cytauxzoon* spp. Tick-borne pathogens in cats have come into the spotlight during the last years, due to a re-evaluation of the clinical impact, apparent emergence, and prevalent occurrence, along with new data on epizootiological and zoonotic potential of some of them [105,106,107,108,109]. However, as ectoparasites were out of the scope of the present study, no further analysis of the ticks was made.

### 4.8. Polyparasitism and Pseudoparasitism

As expected, mixed parasitoses and polyparasitism were found in the present investigation in all animals and almost all faecal samples. The various combinations of mixed infections (Table 3) depict the high biodiversity of wildcats’ parasitic fauna and reflect the various transmission patterns to which wildcats are subjected. This could also be the result of mixed infections in paratenic and intermediate hosts, which are important for bridging infections between wild and domestic animals. Indeed, mixed infections with felid lungworms have been documented in gastropod intermediate hosts in Greece [110]. Depending on various factors, e.g., overall health status, environmental conditions, and food availability, wildlife may maintain an equilibrium with their parasitic fauna. Nonetheless, this parasite/s–host relationship is fragile, and under certain circumstances, the health and life of infected animals may be compromised and threatened [12]. Common factors that may play a role in the onset of the development of a clinical condition due to parasitoses are extreme weather conditions, food scarcity, concomitant infections, environment pollutants, stress, and injuries [8,9,10]. Furthermore, fitness impairment, with no obvious accompanying clinical signs, especially in animals with polyparasitism likely takes place, as for instance occurs in livestock where productivity is greatly impacted by subclinical parasitism [111].

Pseudoparasitism, i.e., the detection of parasite eggs, oocysts, and larvae originating from a prey and not from a true parasitism of the examined animal, is a phenomenon that may complicate copromicroscopic diagnosis of parasitoses in free-ranging carnivores. Thus, careful microscopic examination and a conservative interpretation of findings that could originate from prey is imperative when samples from wildlife and/or predators are examined. For example, in the present study, some findings clearly originated from rodents and/or birds, e.g., Anoplocephalidae and *Trichuris* eggs and *Eimeria* oocysts, while others, e.g., *D. dendriticum*, eggs cannot be excluded from actual infection, although it is more likely to have been derived from infected prey such as lagomorphs, hedgehogs, or rodents. Similarly, in 29 (34.1%) of the 85 of the faecal samples (including field and necropsy-derived samples), mites of the genus *Demodex*, morphologically compatible with *Demodex gatoi* were found. Nonetheless, considering the morphological variability of *Demodex* species found in rodents [112], no definitive conclusions can be drawn about the factual presence of *D. gatoi* in the examined wildcats, although it is possible.

## 5. Conclusions

A wide range of parasites and a high prevalence of infections were evidenced in this survey on the parasitic fauna of wildcats in Greece. Many of the recorded parasites have a significant pathogenic potential, and all of them may cause severe to hazardous diseases of the infected animals. Polyparasitism is a factor that puts at risk the health and welfare of wildcats and, in combination with other natural or anthropogenic factors, may threaten already vulnerable populations. Many parasites recorded in wildcats in the present study may also infect domestic cats with varying rates and frequency. Additionally, some are of zoonotic importance and can threaten human health. Given the environment degradation and habitat loss that enforces wild animals to closer proximity to humans and domestic animals, and under the perspective of the climate change that has been shown to promote many parasitic infections [113], a strategic, constant surveillance of parasites circulating in wildcat populations is imperative, towards (i) an effective protection of wildlife populations, (ii) a reliable minimisation of the risk of infections for domestic animals, and (iii) a timely diagnosis of human infections.

## Figures and Tables

**Figure 7:**
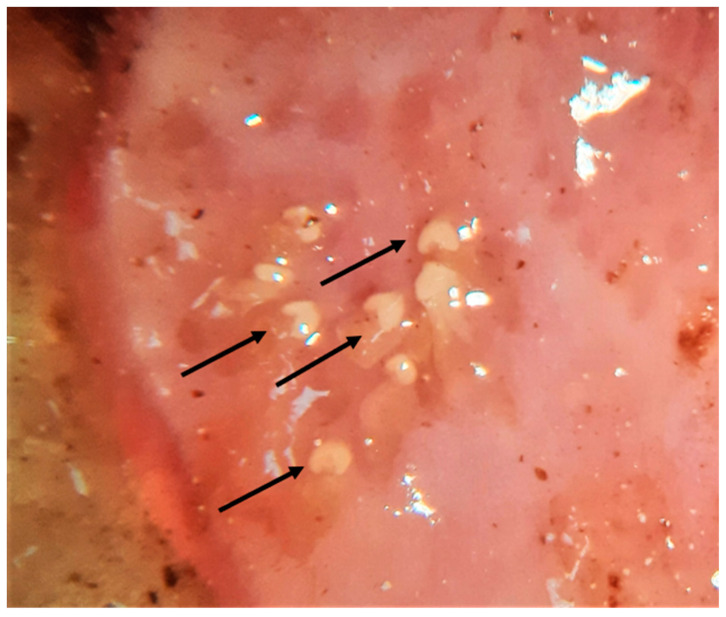
*Alaria alata* adult trematodes (arrows) embedded in the intestinal mucosa of a wildcat.

**Figure 9:**
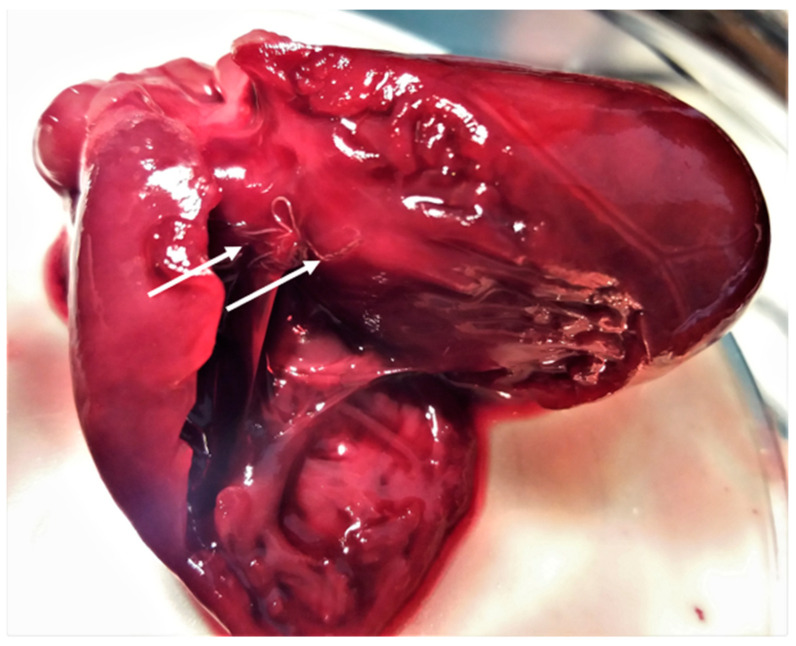
*Angiotrongylus chabaudi* adults (arrows) found in the right chambers of the heart in a wildcat.

**Figure 10:**
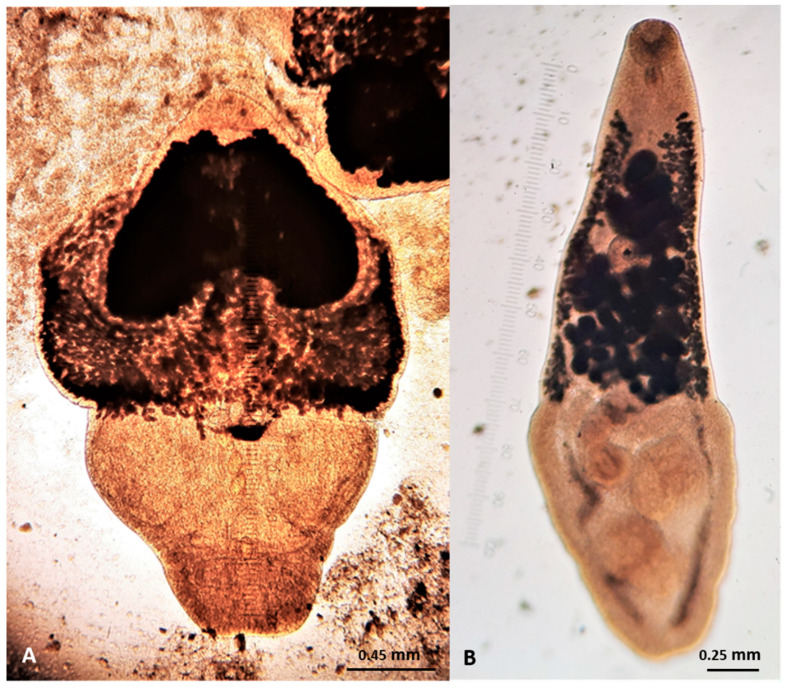
Adult trematodes found in wildcats in Greece. (**A**) *Alaria alata*. (**B**) *Opisthorchis felineus*.

**Figure 11:**
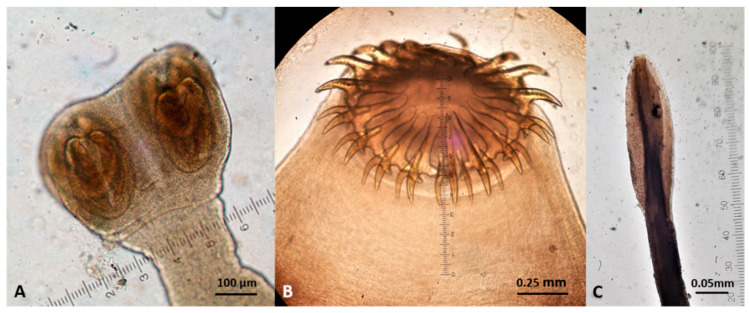
Cestodes (scoleces) found in wildcats in Greece. (**A**) *Mesocestoides* spp. (**B**) *Taenia taeniaeformis*. (**C**) *Spirometra* spp.

**Figure 12:**
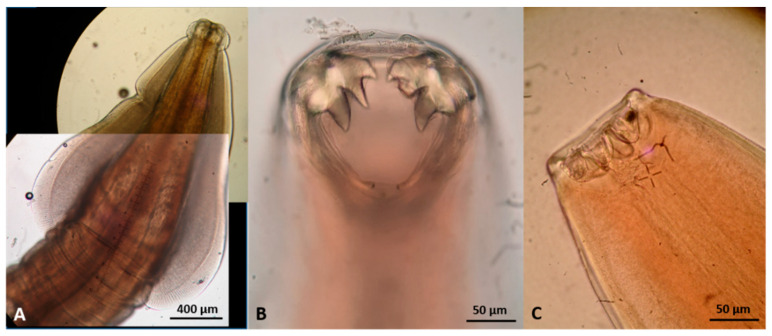
Gastrointestinal nematodes (anterior end) found in wildcats in Greece. (**A**) *Toxocara cati*. (**B**) *Ancylostoma tubaeforme*. (**C**) *Cylicospirura petrowi*.

**Figure 13:**
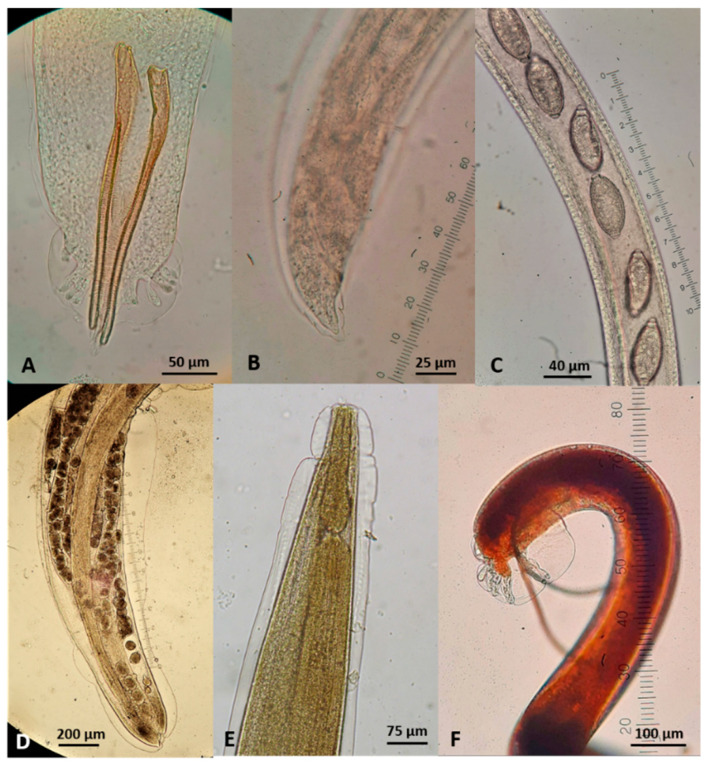
Extra-intestinal nematodes found in wildcats in Greece. (**A**) *Aelurostrongylus abstrusus* male, posterior end. (**B**) *A. abstrusus* female, posterior end. (**C**) *Capillaria aerophila* female, part of the body showing uterus filled with eggs. (**D**) *Oslerus rostratus* female, posterior end. (**E**) *Troglostrongylus brevior* anterior end. (**F**) *T. brevior* male, posterior end.

**Figure 14:**
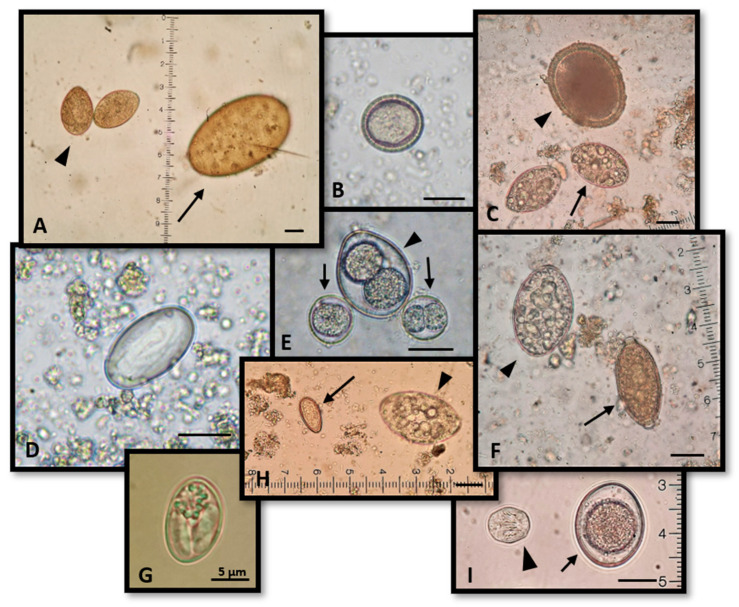
Representative findings of parasite reproductive elements in wildcats in Greece. (**A**) *Spirometra* spp. (arrowhead) and *Alaria alata* (arrow) eggs. (**B**) *Taenia taeniaeformis* egg. (**C**) *Toxocara canis* (arrowhead) and *Spirometra* spp. (arrow) eggs. (**D**) *Cylicospirura* spp. egg. (**E**) *Cystoisospora rivolta* (arrows) and *Cystoisospora felis* (arrowhead) oocysts. (**F**) *Spirometra* spp. (arrowhead) and *Capillaria aerophila* (arrow) eggs. (**G**) *Sarcosystis* spp. sporocyst. (**H**) *Opisthorchis felineus* (arrow) and *Spirometra* spp. (arrowhead) eggs. (**I**) *Mesocestoides* spp. egg (arrowhead) and *Isospora felis* oocyst (arrow). Scale: 20 μm unless differently noted.

**Table 1:** Parasites found in wildcats (faecal samples and road killed animals) in Greece.

Higher Taxon	Parasite	Positive Cases (%) in Faecal Examinations	Positive Cases (%) in Necropsy	Positive Cases (%) in Faeces of Necropsied Animals	Positive Cases (%) in Road Killed Animals (Necropsy Combined with Faecal Examination)
Protozoa	*Cystoisospora felis*	8 (12.9)		6 (26.1)	6 (26.1)
	*Sarcocystis* spp.	10 (16.1)		1 (4.3)	1 (4.3)
	*Cystoisospora rivolta*	3 (4.8)		3 (13)	3 (13)
	*Toxoplasma*/*Besnoitia*/*Hammondia*	1 (1.6)		1 (4.3)	1 (4.3)
Trematoda	*Alaria alata*	5 (8)	4 (17.4)	4 (17.4)	4 (17.4)
	*Opisthorchis felineus*	1 (1.6%)	1 (4.3)	3 (13)	3 (13)
	Trematode eggs	3 (13)		2 (8.7)	2 (8.7)
Cestoda	*Taenia taeniaeformis*	1 ^1^ (1.6)	17 (73.9)	4 (17.4)	17 (73.9)
	*Mesocestoides* spp.		4 (17.4)		4 (17.4)
	Diphyllobothriidae eggs	8 (12.9)		5 (21.7) ^2^	
	*Spirometra* spp.		4 (17.4)		4 (17.4)
Nematoda	*Toxocara cati*	28 (45.2)	13 (56.5)	9 (39.1)	14 (60.9)
	*Capillaria aerophila*	15 (24.2)	6 (26.1)	7 (33.8)	7 (33.8)
	*Angiostrongylus chabaudi*	18 (29)	13 (56.5)	11 (50%)	13 (56.5)
	*Aelurostrongylus abstrusus*	6 (9.7)	4 (17.4)	10 (43.5)	10 (43.5)
	*Ancylostoma tubaeforme*	11 ^1^ (17.7)	9 (39.1)	7 (33.8)	9 (39.1)
	*Troglostrongylus brevior*	6 (9.7)	8 (34.8)	7 (33.8)	8 (34.8)
	Capillariidae eggs	6 (9.7)		3 (13)	
	*Cylicospirura* spp.	4 (6.25)	8 (34.8)	4 (17.4)	8 (34.8)
	*Physaloptera* spp.	1 (1.6)	4 (17.4)	1 (4.3)	4 (17.4)
	*Thelazia callipaeda*		2 (8.7)		2 (8.7)
	*Oslerus* spp.		1 (4.3)		1 (4.3)
	*Toxascaris leonina*		1 (4.3)	1 (4.3)	1 (4.3)
	*Dirofilaria immitis*		1 (4.3)		1 (4.3)
Acanthocephala	*Centrorynchus* spp.		1 (4.3)		1 (4.3)
Arthropoda	Ixodidae		4 (17.4)		4 (17.4)

^1^ eggs identified to the corresponding family level. ^2^ corresponding to *Mesocestoides* spp. and *Spirometra* spp.

**Table 2:** Number of parasite taxa per higher taxon identified in each of the necropsied wildcats.

Wildcat	Protozoa	Trematoda	Cestoda	Nematoda	Acanthocephala	Arthropoda	Total
1	-	1	-	2	-	-	3
2	3	-	-	4	-	-	7
3	2	-	2	5	-	-	9
4	1	1	1	5	1	1	10
5	-	1	1	3	-	-	5
6	-	-	1	5	-	-	6
7	-	-	2	5	-	1	8
8	-	-	1	4	-	-	5
9	-	-	1	7	-	1	9
10	1	-	1	6	-	1	9
11	-	1	2	6	-	-	9
12	-	-	1	8	-	-	9
13	-	-	-	4	-	-	4
14	-	-	2	2	-	-	4
15	2	1	1	4	-	-	8
16	1	-	1	1	-	-	3
17	1	1	2	3	-	-	7
18	-	-	2	4	-	-	6
19	-	1	-	4	-	-	5
20	-	-	1	3	-	-	4
21	-	-	1	2	-	-	3
22	-	-	1	2	-	-	3
23	-	-	1	1	-	-	2

**Table 3:** Combinations of taxa in mixed parasitic infections found by necropsy in wildcats in Greece.

Infection	n/tot (%)
Cestoda + Nematoda	9/23 (39.1)
Protozoa + Cestoda + Nematoda	2/23 (8.7)
Trematoda + Cestoda + Nematoda	2/23 (8.7)
Cestoda + Nematoda + Arthropoda	2/23 (8.7)
Protozoa + Trematoda + Cestoda + Nematoda	2/23 (8.7)
Protozoa + Trematoda + Cestoda + Nematoda + Acanthocephala + Arthropoda	1/23 (4.3)
Protozoa + Cestoda + Nematoda + Arthropoda	1/23 (4.3)
Protozoa + Nematoda	1/23 (4.3)
Trematoda + Cestoda	1/23 (4.3)
Trematoda + Nematoda	1/23 (4.3)
Nematoda	1/23 (4.3)

## Data Availability

No new data were created for this review article.
